# Lytic polysaccharide monooxygenases and other histidine-brace copper proteins: structure, oxygen activation and biotechnological applications

**DOI:** 10.1042/BST20201031

**Published:** 2021-01-15

**Authors:** Johan Ø. Ipsen, Magnus Hallas-Møller, Søren Brander, Leila Lo Leggio, Katja S. Johansen

**Affiliations:** 1 Department of Plant and Environmental Sciences, Copenhagen University, Frederiksberg DK-1871, Denmark; 2 Department of Geosciences and Natural Resource Management, Copenhagen University, Frederiksberg DK-1958, Denmark; 3 Department of Chemistry, University of Copenhagen, DK-2100 Copenhagen Ø, Denmark

**Keywords:** bioethanol, copper, lignocellulose, LPMO

## Abstract

Lytic polysaccharide monooxygenases (LPMOs) are mononuclear copper enzymes that catalyse the oxidative cleavage of glycosidic bonds. They are characterised by two histidine residues that coordinate copper in a configuration termed the Cu-histidine brace. Although first identified in bacteria and fungi, LPMOs have since been found in all biological kingdoms. LPMOs are now included in commercial enzyme cocktails used in industrial biorefineries. This has led to increased process yield due to the synergistic action of LPMOs with glycoside hydrolases. However, the introduction of LPMOs makes control of the enzymatic step in industrial stirred-tank reactors more challenging, and the operational stability of the enzymes is reduced. It is clear that much is still to be learned about the interaction between LPMOs and their complex natural and industrial environments, and fundamental scientific studies are required towards this end. Several atomic-resolution structures have been solved providing detailed information on the Cu-coordination sphere and the interaction with the polysaccharide substrate. However, the molecular mechanisms of LPMOs are still the subject of intense investigation; the key question being how the proteinaceous environment controls the copper cofactor towards the activation of the O-O bond in O_2_ and cleavage of the glycosidic bonds in polysaccharides. The need for biochemical characterisation of each putative LPMO is discussed based on recent reports showing that not all proteins with a Cu-histidine brace are enzymes.

## Introduction

The replacement of liquid fossil transportation fuels with ethanol produced from agricultural and forest lignocellulosic residues is now technically possible [1]. However, the first commercial lignocellulosic biorefineries have suffered competition from historically low crude oil prices, which has made them economically strained. Although the biotechnological aspects of the process are in place, further improvements are required in process economy. Efficient saccharification takes place during incubation of the lignocellulosic material with the enzyme cocktail, at a slightly elevated temperature (typically 50°C) and constant pH (typically around pH 5), while the mixture is stirred. Enzyme cocktails consisting of many different types of enzymes are required for saccharification of the complex polysaccharide components of lignocellulose. When bioethanol is the desired product, yeast is added after 3–5 days of incubation to ferment the released glucose and xylose to ethanol. The residual material is high in lignin, and can be incinerated to produce steam and electricity [1]. Alternatively, it could potentially be used to produce marine fuel [2].

Lignocellulose is a complex recalcitrant matrix dominated by lignin, cellulose and hemicellulose, which provides the plant tissue with strength and durability. The most abundant polysaccharide in lignocellulose is cellulose, and several enzymes are involved in its decomposition into glucose. The process requires the synergistic action of endo-acting glycosyl hydrolases (endoglucanase), processive exo-hydrolases acting from the reducing end (cellobiohydrolase I), and processive exo-hydrolases acting from the non-reducing end (cellobiohydrolase II) of the cellulose chains. The overall reaction is driven towards completion by β-glycosidases through the alleviation of cellobiose product inhibition of the exo-enzymes [3–5]. Despite early reports indicating that an oxidative enzymatic step was crucial for cellulose saccharification [6–8], this notion was not fully accepted until the discovery of lytic polysaccharide monooxygenases (LPMOs) [9,10]. LPMOs initiate the saccharification of cellulose by oxidative cleavage of internal glucosidic bonds that are not accessible to the endo-acting hydrolases. LPMOs are mononuclear copper enzymes that are particularly common in the secretomes of saprophytic fungi feeding on lignocellulosic materials such as wood. This review discusses the Cu-active site of LPMOs, and how protein structure together with oxygen activation contribute to a catalytic cleavage.

## LPMOs are classified into several enzyme families

LPMOs are classified in the Carbohydrate-Active EnZymes database (CAZy). They are grouped in enzyme families termed Auxiliary Activities (AAs) that catalogue the redox enzymes involved in carbohydrate degradation [11]. LPMOs have been demonstrated to have substrate competency towards a diverse group of polysaccharides, for example starch, xylan, xyloglucans and other hemicelluloses [12–16]. However, the two most studied activities are those on cellulose and chitin. Based on sequence similarity LPMOs are currently classified as AA9–AA11 and AA13–AA16 in the CAZy database. The most well characterised LPMOs are from the families AA9 and AA10, with multiple examples of biochemical characterisations and entries in the Protein Data Bank (PDB). While the first LPMOs were identified through fractionation of fungal secretomes in combination with saccharification assays [17], newer LPMOs have often been identified by data mining of genomic sequences. AA14 has recently been defined as a LPMO family, based on the characterisation of two enzymes from the white-rot basidiomycete *Pycnoporus coccineus* (now *Trametes coccineus*) [18]. The two *T. coccineus* enzymes demonstrated no enzymatic activity when used alone on 11 different polysaccharides, however, when incubated with either different *Trichoderma reesei* enzyme cocktails or with a GH11 xylanase, a synergistic boost was observed in the saccharification of xylan bound to cellulose in woody biomasses. Since no LPMO activity could be demonstrated on isolated polysaccharides, more detailed biochemical characterisation of the two enzymes is needed to deduce their enzymatic mechanisms. Interestingly, the presence of LPMOs in nature has recently expanded outside microorganisms, with examples being found in insects, animals and ferns [19,20]. The AA15 family has been discovered in the gut of the insect *Thermobia domestica* [20]. Biochemically characterised members of the AA15 family were found to degrade both cellulose and chitin. The most recent addition is the AA16 family, where an LPMO from the ascomycete *Aspergillus aculeatus* was heterologously expressed and biochemically shown to oxidatively cleave cellulose at the C1 position [21]. However, the amount of product formed was very low, and more thorough biochemical characterisation and its crystal structure are required to elucidate the enzymatic nature of this LPMO family.

In 2016, a protein (Tma12) with high sequence similarity to LPMOs from the AA10 family was identified in the fern *Tectaria macrodonta* [22]. Tma12 was identified through purification of extracts from fronds coupled with a reverse mass spectrometry approach. Interestingly, heterologous expression of Tma12 in cotton conferred an increased resistance to whitefly infection. This observation suggests a similar insecticidal effect of the *T. macrodonta* LPMO as the putative LPMO from the insect poxvirus [23]. The crystal structure was solved and showed structural similarity to cellulolytic LPMOs [19]. This indicates that LPMOs can be found in at least one branch of the plant kingdom.

## LPMOs contain a Cu-histidine brace motif

**Figure 1.**
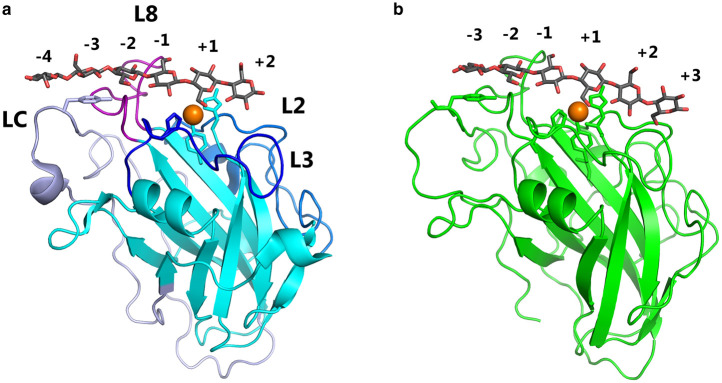
Structures of two LPMOs in complex with their substrate. Interactions of *Ls*AA9A (**a**) and CvAA9A (**b**) in crystal complexes with cellohexaose (PDB codes 5ACI and 6YDE [29,33]). Substrate-binding subsites are indicated by numbers. The active site copper is shown as an orange sphere, with interacting residues as sticks. The Tyr from the LC loop interacting with the substrate at the −3 subsite is also shown. Hydrogen bonding residues from the L2, L3 and L8 loops are not shown for clarity. Loops are indicated on the *Ls*AA9A structure. Protein structures were visualised using PyMOL 2.4.

The main feature of the LPMO structure is the metal-binding site, composed of two histidine residues exposing the bound Cu atom to the environment, situated in the middle of one of the flat surfaces of the protein later shown to bind substrate (Figure 1). This is the Cu-histidine brace motif. The two nitrogen atoms from the histidine side-chains and a third from the N-terminus are all interacting with the Cu-atom at a distance of close to 2 Å [9,24].

One of the first characterised LPMOs was found in *Thermoascus aurantiacus* (*Ta*AA9A). The affinity for Cu(II) was found by isothemal titration calorimetry (ITC) to be in the low nanomolar range, this was confirmed using electron paramagnetic resonance (EPR), which suggested a dissociation constant (*K*_d_) in the pM range [9]. Similar direct determination of affinity has been carried out for an AA10 member from *Serratia marcescens* (*Sm*AA10A). Interestingly, *Sm*AA10A was found to bind Zn(II) in addition to Cu(II). The experimentally determined *K*_d_ of 55 nM for Cu(II)-*Sm*AA10A was used to estimate *K*_d_ of 1.2 nM for Cu(I)-*Sm*AA9A [25]. This study clearly showed that LPMOs have a higher affinity for the reduced metal Cu(I). The fact that certain LPMO families can bind Zn(II) was utilised to accurately determine the affinity of *Aspergillus oryzae* AA11 (*Ao*AA11) towards copper. Displacement ITC using (Zn(II)) as the weak ligand to be displaced by the stronger ligand (Cu(II)), found a *K*_d_ of 0.7 nM for *Ao*AA11 [26] and of 43 nM for *Bacillus amyloliquefaciens* AA10 [27]. Taken together, these studies demonstrate a very high affinity of LPMO families towards copper. Interestingly, it has been demonstrated that LPMOs have an increased affinity for polysaccharide substrate when reduced [28]. This could mean that the reduction in the metal not only primes the enzyme for catalysis, but also directs it towards its substrate by increased affinity.

## Structural features of LPMOs

Determination of the structures of LPMOs has contributed considerably to our understanding of LPMOs (detailed structural reviews can be found in [29–31]). To date, structures are available for over 40 different LPMOs, belonging primarily to the AA9 and AA10 families, and one representative each of the AA11, AA13, AA14 and AA15 families. Common to all LPMOs is a β-sandwich core structure consisting of two β-sheets comprising seven or eight β-strands in total. Structural diversity is generated by the helices and loops that connect the core β-strands, giving rise to variations in the dimensions and topologies of the substrate-binding surface. Fungal LPMOs are often methylated at the N-terminal histidine. The role of this methylation was recently investigated through parallel assays of *Ta*AA9A expressed in *Pichia pastoris* (unmethylated) and *Aspergillus oryzae* (methylated), and it was suggested that methylation is involved in protection against auto-oxidative inactivation of the LPMO [32]. The reason why methylation is only observed in LPMOs expressed in filamentous fungi is not known.

## The interaction between LPMOs and their polysaccharide substrate

When the first structures of fungal LPMOs in the AA9 family were determined, it became apparent that they shared a striking structural similarity to certain carbohydrate-binding modules (CBMs) [17,34,35]. This confirmed a connection between the AA9 and AA10 families (formerly GH61 and CBM33). Some features were lacking compared with glycoside hydrolases, most notably a clear active site cleft or groove. These absences pointed towards a different reaction mechanism than the one identified in glycoside hydrolases. However, some similarities were found as an AA9 LPMO from the ascomycete *Thielavia terrestris* (now *Thermothielavioides terrestris*) showed an arrangement of aromatic residues that was strongly reminiscent of a carbohydrate binding module with specificity for crystalline polysaccharides [36], suggesting that these proteins acted by binding to crystalline cellulose.

It has been difficult to obtain detailed structural information on the interaction of LPMOs with their polysaccharide substrates [13,37]. However, X-ray crystallographic, nuclear magnetic resonance (NMR), EPR studies, and computational studies have given us some insights into the process. Interaction studies by crystallography are only possible on LPMOs able to bind relatively small fragments (oligosaccharides), and it has so far only been successful with two AA9 enzymes that are able to cleave oligosaccharides by C4 oxidation [29,33,38]. In addition to the His-brace region, four loops contribute residues interacting with the substrate, mostly through hydrogen bonds, but also with a highly conserved aromatic residue (Tyr203 in *Ls*AA9A, Figure 1a). These studies have provided a very detailed picture of the interactions of the active sites of these LPMOs with their oligosaccharide substrates. Unfortunately, these insights are not directly transferable to LPMOs acting on crystalline polysaccharides.

Another experimental technique that can provide structural information on the interaction of LPMOs with substrates is NMR spectroscopy, which can be used to identify protein residues interacting with the polysaccharide through changes in chemical shifts, as carried out for both AA9 and AA10 LPMOs [25,39]. However, NMR is only suitable for diamagnetic species, and the paramagnetic Cu(II) will distort signals from all nearby nuclei. Thus, this technique is useful when the LPMO is in e.g. Cu(I), Zn(II) or apo-form. While NMR spectroscopy cannot provide the same level of detail as X-ray crystallography, it has the advantage that interactions with both large polysaccharide substrates and small oligosaccharides can be probed.

EPR spectroscopy takes advantage of the paramagnetic properties of Cu(II), and has been used extensively in LPMO research to probe the active-site copper. EPR spectroscopy has recently been utilised to provide experimental information in the modelling of interactions between LPMO and oriented celery cellulose fibres [40], confirming previous suggestions that *Ls*AA9A attacks the edge of cellulose fibres, rather than the flatter surfaces. Surprisingly, it was concluded that *Ta*AA9A also attacks the same edge, which is somewhat counterintuitive given the rather different shapes of the binding surfaces of these two LPMOs.

Computational studies (docking and molecular dynamics) have been carried out to investigate the interactions of LPMOs with crystalline polysaccharides; for example, an extensive study on chitinolytic AA10 [41], and the recent study on a cellulose-specific LPMO [42], in both cases showing interactions with the flat faces of crystalline polysaccharides.

## LPMOs use the oxidative power of oxygen

**Figure 2.**
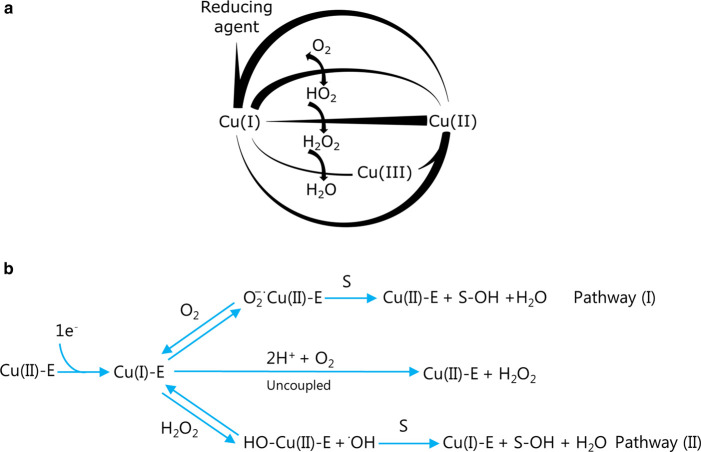
Simplified reaction schemes for free copper and Cu-LPMO. (**a**) Free copper reactions based on [44] (**b**) Cu-LPMO reactions based on [31,48]. Pathway I: O_2_ binds to the Cu(I)-active site of the Enzyme (E). In the presence of a polysaccharide substrate (S), O_2_ reduction leads to substrate hydroxylation. In the absence of a polysaccharide, O_2_ reduction produces H_2_O_2_. Pathway II: H_2_O_2_ generated *in situ* can react with the Cu(I)-active site. When the polysaccharide is bound, substrate oxidation occurs. Small-molecule reductants or enzymes, such as cellobiose dehydrogenase, can provide the necessary reducing equivalents.

LPMOs have been classified as oxidases by the International Union of Biochemistry and Molecular Biology, and their catalytic reaction with O_2_ and polysaccharides gives the numbers EC 1.14.99.53 (chitin), EC 1.14.99.54 (C1 dehydrogenation on cellulose), EC 1.14.99.56 (C4 dehydrogenation on cellulose), and EC 1.14.99.55 (starch). However, the current understanding of the catalytic competencies of LPMOs is far more complex and is briefly discussed here. Figure 2a illustrates Cu-catalysed reactions coupled to the reduction in O_2_ to water at circumneutral pH [43,44]. The reduction in O_2_ to superoxide is thermodynamically disfavoured, and only trace amounts of superoxide are produced. Superoxide is reduced by a fast and efficient reaction with Cu(I) to form H_2_O_2_, and it is this fast reduction in superoxide that drives the reduction in O_2_ by virtue of Le Chatelier's principle. The surface-exposed Cu-histidine brace of LPMOs can take part in similar chemical reactions. The simplified LPMO reaction scheme shown in Figure 2b illustrates this point well. In pathway I, the resting-state LPMO (Cu(II)-E) is first reduced by one electron to Cu(I)-E, which is re-oxidised by O_2_. The reaction has been experimentally demonstrated, and the re-oxidation rate found to differ 10 fold between two LPMOs [45,46]. An uncoupled reaction that can be detected by the production of H_2_O_2_ takes place in the absence of a polysaccharide substrate [45,47–49]. The mechanism behind the uncoupled reaction has been investigated using quantum mechanics/molecular mechanics simulations [50,51]. In the presence of a polysaccharide substrate the monooxygenase reaction takes place, and oxidised saccharide products and water are produced, while returning the enzyme to the resting state Cu(II)-E. This was first demonstrated using ^18^O_2_ for the chitin-active *Sm*AA10A [10], and confirmed for a cellulose-active LPMO from *Neurospora crassa* (NCU08760) [52]. In pathway II, H_2_O_2_ reacts as the co-substrate with Cu(I)-E in a reaction that produces oxidised saccharide products, water and the reduced enzyme. The H_2_O_2_ may be from an external source or it may be produced by the LPMOs in the uncoupled reaction. Pathway II was first suggested in 2017 [49] and examined with a cellulolytic AA9 enzyme from *Hypocrea jecorina* [46]. The re-oxidation rate of Cu(I)-LPMO with H_2_O_2_ was found to be 1,000 times faster than with O_2_ [46,53]. The reaction path with H_2_O_2_ is likely branched, but homolytic cleavage of H_2_O_2_ has been identified as the predominant path based on single-turnover experiments [46].

As described above, LPMOs bind poly- or oligosaccharides in a fixed position with no distortion of the substrate across the Cu-histidine brace [29]. It has been suggested that the binding of a polysaccharide and a co-substrate may be synergistic [29], random-sequential [48] or ordered-sequential [46]. Cleavage of the glycosidic bonds is believed to start with the abstraction of a hydrogen atom from carbon number 1 or 4 of the sugars (C1 or C4) on either side of the bond. This is a chemically difficult reaction because of the C-H bond strength of nearly 100 kcal/mol [54]. The regiospecificity (i.e. the propensity of the products to be oxidised at C1 or C4) is not strictly associated with AA families, and can be substrate-dependent [38,54,55]or reductant-dependent [56]. Although several mechanisms have been proposed, and some examined computationally [57], the mechanistic details of the reaction(s) leading to the cleavage of glycosidic bonds are still unknown.

## LPMOs in biotechnological applications

While pure cellulose is not degraded by LPMOs in classic cellulase assays, it is clear that lignocellulose is [9]. The liquid fraction from pretreated lignocellulose, such as straw, contains different low-molecular-weight compounds that instigate activity. Many reducing agents, such as gallic acid [9], ascorbate, cysteine [18] and others [12,58] will have the same effect, and have been used in laboratory experiments. However, the relationship between reducing agents (including oxidoreductases and metals [9,14,52,59,60]), LPMOs and oxygen during the saccharification of lignocellulose is complicated [61,62]. Our own studies have shown that the industrial decomposition of lignocellulose takes place at low levels of dissolved O_2_, due primarily to abiotic O_2_-consuming reactions [61,62]. Importantly, we found that even when the lignocellulosic material was flushed with only 2% O_2_ (compared with air), reactive oxygen species had to be removed by catalase in order to stabilise the enzyme cocktail. This finding implies that H_2_O_2_ is generated during incubation of the material. As discussed above, H_2_O_2_ has been shown to function as a co-substrate in the place of O_2_ in a rapid catalytic cycle [53]. It was recently shown that the liquid fraction of pretreated wheat straw could provide both electrons and H_2_O_2_ for LPMO catalysis under oxic conditions [63]. Evidence suggests that while H_2_O_2_ leads to faster initial rates of polysaccharide oxidation than O_2_ [46], it also reduces the half-life of the enzyme cocktail in industrially relevant assays [61]. Furthermore, oxic conditions also resulted in acidification and decarboxylation of the lignocellulosic material by chemical reactions that are affected by oxidoreductases in the mixture [62]. Abiotic and enzymatic oxidative processes are thus intertwined during the decomposition of plant materials, and transition metals, Cu in particular, are involved in both.

LPMOs and the industrial use of these enzymes have been described in the patent literature. A large proportion of patent applications describe technical advances of relevance to biotechnological processes involving plant material. The use of LPMOs for the decomposition of cellulose [17] and starch [64] are good examples thereof. In another example, the inventors claimed the use of an AA9 LPMO that has activity on xylan [65]. Xylans are major components of hemicellulose involved in the integrity of the plant cell wall. The removal of xylans increases the productivity of commercial enzymes, likely by increasing substrate accessibility [66]. LPMOs may also improve the fermentation of starch-derived sugars to desirable products by reducing the formation of lactic or acetic acidic acid during incubation [67]. Several patent applications that mainly disclose DNA sequences of several amino acid variants of LPMOs with desirable qualities have been published, a recent example being that by Lin et al. [68]. These are a few examples from the patent landscape that document the broad commercial interest and potentially significant economic gain of using LPMOs as biotechnological process tools.

## LPMO-like proteins and other proteins that share structural features with LPMOs

**Figure 3.**
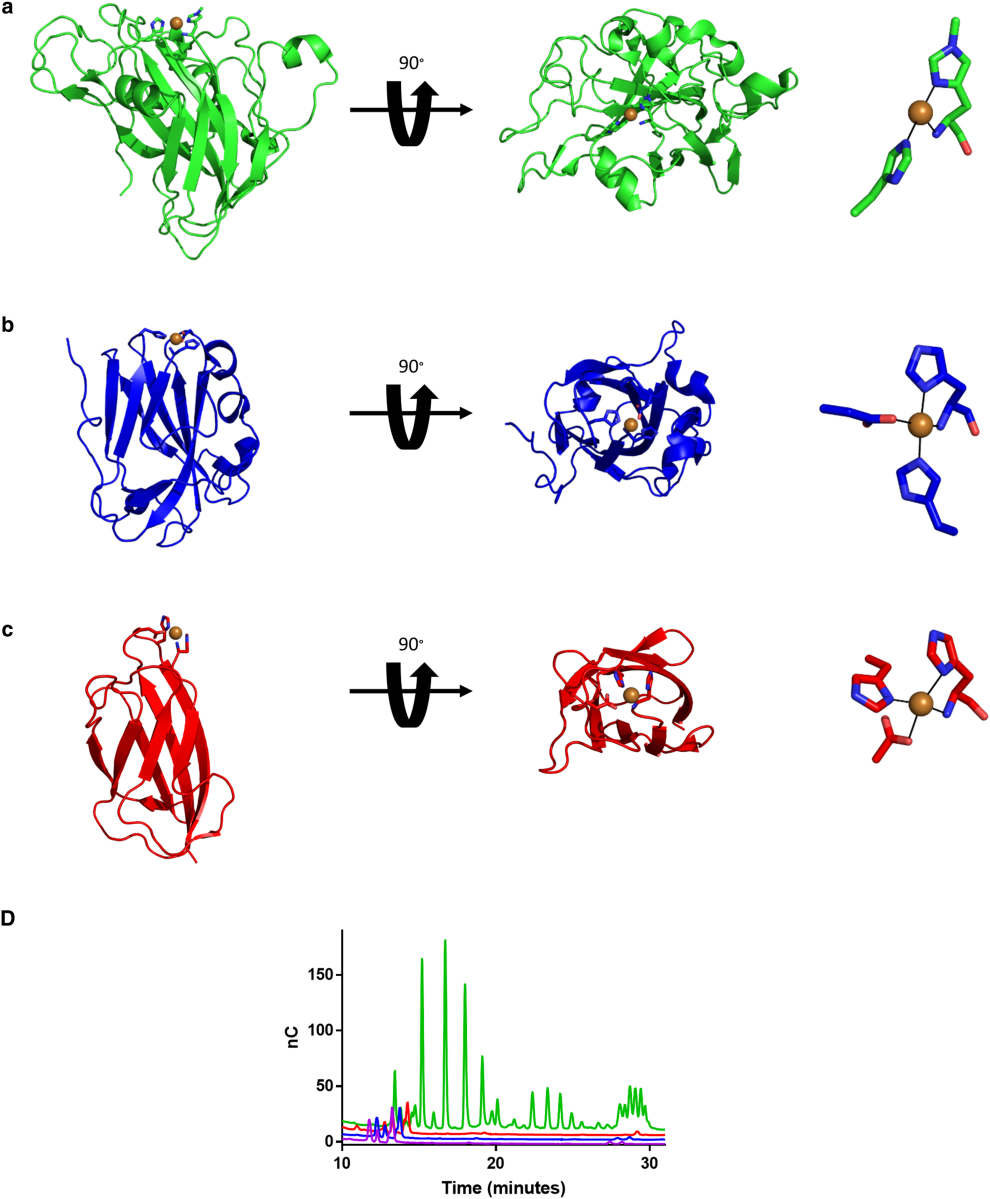
Three protein folds all containing the Cu-histidine brace. A(**a**) Representative cellulose-active LPMO, *Ta*AA9A (green) (PDB: 2YET) [9]. (**b**) The LPMO-like protein *La*X325 (blue) (PDB: 6IBJ) from *Laetisaria arvalis* which, based on sequence and predicted fold, belongs to the same family of LPMO-like proteins as Bim1 [37]. (**c**) The Cu chaperone, CopC, from *Pseudomonas fluorescence* (red) (PDB: 6NFQ) [75]. (**d**) Results of HPAEC-PAD analysis of the three proteins when incubated with phosphoric acid swollen cellulose and ascorbate for 24 h [44]. Only the LPMO trace (green) shows polysaccharide-degrading activity. The purple trace is that of aqueous copper. Protein structures were visualised using PyMOL 2.4.

As discussed above, the field of LPMO research has historically had a strong focus on enzymes with potential to be applied in biomass reactors. The structure of a model cellulose active LPMO *Ta*AA9A is shown in Figure 3a. More recently, the field has turned towards exploring the roles and diversity of these enzymes in nature. However, the identification of LPMOs has proven to be more difficult than first expected. One attempt to identify novel fungal LPMOs instead revealed a family of LPMO-like proteins (termed X325). The X-ray structure of a member of the X325 family from the ectomycorrhizal fungus *Laccaria bicolor* has been solved (Figure 3b) [37]. Although the structure displays a His brace Cu-binding site on the protein, no polysaccharide-degrading activity has been detected for several members of X325 [37,69]. One member of the X325 family has been identified in the human pathogen *Cryptococcus neoformans*. The expression of this protein, called Bim1, was seen to dramatically increase under Cu-limiting conditions. Bim1 participates in Cu uptake in concert with the Cu(I) importer Ctr1, and is a critical factor for Cu acquisition in fungal meningitis [69]. Intriguingly, a similar Cu-His-brace motif has also been found in the small bacterial periplasmic protein CopC (Figure 3c), which also functions in copper homeostasis and the delivery of copper to specific transporters. In a recent study, we compared the biochemical properties of CopC from *Pseudomonas fluorescens* with Bim1 and the well-described cellulose-specific *Ta*AA9A [44]. Only *Ta*AA9 was found to cleave cellulose (Figure 3d) as was expected because it contains an appropriate polysaccharide binding site while the others do not. However, the CopC protein is inert to the relevant reductant ascorbate [44] and thus unable to initiate the cycle between redox states that is associated with LPMO activity. This confirms that CopC is not an LPMO despite the presence of a Cu histidine brace. The importance of some first and second sphere residues in LPMOs for activity has been investigated experimentally [17,70] and computationally [57,71]. These important residues could explain the differences in copper reactivity. For a more in depth review of the hydrogen bonding network and implications of the second coordination sphere see [72].

Another Cu-histidine brace found in nature is the B-site in particulate methane monooxygenases. These enzymes catalyse the oxidation of methane to methanol. Until recently, this site was identified as the active site, in part due to its similarities to the LPMO histidine brace. However, another mononuclear copper site with different coordination geometry more recently has been suggested to be the active site [73,74]. It is thus clear that biochemical demonstration of substrate cleavage activity is important when claiming redox activity, since both the LPMO fold and Cu-histidine brace motif can adapt to different and diverse functions.

## Perspectives

- Within the field of industrial biotechnology, LPMOs are of the utmost importance for efficient decomposition and processing of plant materials.
- LPMOs and LPMO-like proteins are widely found in nature. The Cu-active site of LPMOs undergoes redox-cycling to catalyse cleavage of polysaccharides using molecular mechanisms that rely on redox partners and use oxygen species as co-substrate.
- Both enzymatic and abiotic oxidative processes must be carefully controlled in biorefineries. Further studies are required to shed further light on how LPMOs acts *in vivo*.

## Abbreviations

CAZy: Carbohydrate-Active EnZymes database
EPR: electron paramagnetic resonance
ITC: isothemal titration calorimetry
LPMOs: Lytic polysaccharide monooxygenases
NMR: nuclear magnetic resonance
PDB: Protein Data Bank
